# Lipoprotein(a) Serum Levels Predict Pulse Wave Velocity in Subjects in Primary Prevention for Cardiovascular Disease with Large Apo(a) Isoforms: Data from the Brisighella Heart Study

**DOI:** 10.3390/biomedicines10030656

**Published:** 2022-03-11

**Authors:** Arrigo F. G. Cicero, Federica Fogacci, Giuseppe Derosa, Angela D’Angelo, Fulvio Ventura, Elisabetta Rizzoli, Sergio D’Addato, Claudio Borghi, on behalf of the Brisighella Heart Study Group

**Affiliations:** 1 Hypertension and Cardiovascular Risk Factors Research Center, Medical and Surgical Sciences Department, Sant’Orsola-Malpighi University Hospital, Via Albertoni 15, 40138 Bologna, Italy; federicafogacci@gmail.com (F.F.); elisabetta.rizzoli@unibo.it (E.R.); sergio.daddato@unibo.it (S.D.); claudio.borghi@unibo.it (C.B.); 2 IRCCS Policlinico S. Orsola—Malpighi di Bologna, 40138 Bologna, Italy; fulvio.ventura90@gmail.com; 3 Department of Internal Medicine and Therapeutics, University of Pavia, 27100 Pavia, Italy; giuseppe.derosa@unipv.it (G.D.); angela.dangelo@unipv.it (A.D.)

**Keywords:** lipoprotein(a), Lp(a), apolipoprotein(a), apo(a), kringles, pulse wave velocity, arterial stiffness

## Abstract

In the last decades, high serum levels of lipoprotein(a) (Lp(a)) have been associated with increased cardiovascular disease (CVD) risk, in particular among individuals with smaller apolipoprotein(a) (apo(a)) isoforms than those with larger sizes. The aim of our analysis was to evaluate whether Lp(a) levels could predict early vascular aging, and whether smaller apo(a) isoforms had a predictive value for vascular aging different than larger apo(a) isoforms in a cohort of subjects free from CVD. We considered the data of a subset of Brisighella Heart Study (BHS) participants free from CVD (462 men and 516 women) who were clinically evaluated during the 2012 BHS population survey. Predictors of arterial stiffness, measured as carotid-femoral pulse wave velocity (cfPWV) were estimated by the application of a step-wise linear regression model. In our cohort, there were 511 subjects with small apo(a) size and 467 subjects with large apo(a) isoforms. Subjects with larger apo(a) isoform sizes had significantly lower serum levels of Lp(a). In the BHS subpopulation sample, cfPWV was predicted by age, systolic blood pressure (SBP), serum levels of high-density lipoprotein cholesterol (HDL-C), triglycerides (TG) and sex, higher HDL-C serum levels and female sex associated with lower values of cfPWV. In subjects with smaller apo(a) isoform sizes, predictors of cfPWV were age, SBP, sex and serum levels of HDL-C, being higher HDL-C serum levels and female sex associated to lower values of cfPWV. In subjects with larger apo(a) isoform sizes, cfPWV was predicted by age, SBP, serum levels of Lp(a) and sex, with female sex associated with lower values of cfPWV. In our subpopulation sample, Lp(a) did not predict cfPWV. However, in subjects with large apo(a) isoform sizes, Lp(a) was a significant predictor of arterial stiffness.

## 1. Introduction

Lipoprotein(a) (Lp(a)) consists of an atherogenic prothrombotic lipoprotein, resulting from the disulfide bond between the Cys4326 in apolipoprotein B100 and the only unpaired cysteine (Cys1568) of plasminogen-like glycoprotein apolipoproteina(a) (apo(a)), in a 1:1 molar ratio [1]. The primary structure of apo(a) has a strictly conserved 80-amino acids sequence—known as ‘kringle’—that is also present in several human proteins involved in a number of vascular functions. In particular, apo(a) consists of multiple copies of plasminogen-like kringle IV (KIV) domains and a single kringle V domain followed by an inactive serine protease domain [2]. Based on the amino acid sequence, the apo(a) KIV domain is subdivided and numbered from type 1–10. KIV type 1 (KIV1) and types 3–10 (KIV3–10) are always present as a single copy, whereas KIV2 occurs in a variable number of identical repeats. Known alleles encode as few as 1 and as many as 34 KIV2 repeats, giving rise to apo(a) isoforms containing from 10 to 43 KIV-like domains [3].

Despite this, there is a relatively large number of studies showing the relationship between Lp(a) serum levels and the risk of non-fatal and fatal cardiovascular (CV) events [4,5], less data are available on the relationship between Lp(a) concentrations and preclinical signs of vascular damage. To date, evidence showing that Lp(a) serum levels are directly related to markers of vascular stiffening refers to small and highly selective groups of patients (e.g., patients with hypertension) [6,7]. However, data referring to population-based cohorts are missing.

In this context, the aim of our study was to evaluate whether serum levels of Lp(a) could predict early vascular aging, measured as carotid-femoral pulse wave velocity (cfPWV). Our analysis also set out to evaluate whether smaller apo(a) isoforms had a predictive value for vascular aging different than larger apo(a) isoforms in a cohort of subjects free from cardiovascular disease (CVD).

## 2. Materials and Methods

### 2.1. Study Design

For this analysis, we used the data of a subset of the Brisighella Heart Study (BHS) participants who were evaluated during the 2012 population survey.

The BHS is a longitudinal epidemiological study started in 1972. At the baseline, the BHS involved a randomized sample of 2939 Caucasian subjects (1448 women and 1491 men), aged 14–84 and free from CVD, that was representative of the entire population of Brisighella, a rural North-Italian village. The study protocol was previously described in detail [8]. Briefly, participants were clinically evaluated in 1972 and every four years from then until now, by performing full clinical examinations and collecting laboratory samples. Specific substudies have been carried out during the period of observation [9].

The study was performed in accordance with the ethical standards promoted by the 1964 Declaration of Helsinki and its later amendments, and its protocol was approved by the Institutional Ethical Board of the University Hospital of Bologna (AVEC; Code: BrixFollow-up_1972-2024). All involved volunteers signed a study-specific informed consent form to participate in to the study.

**Figure 1:**
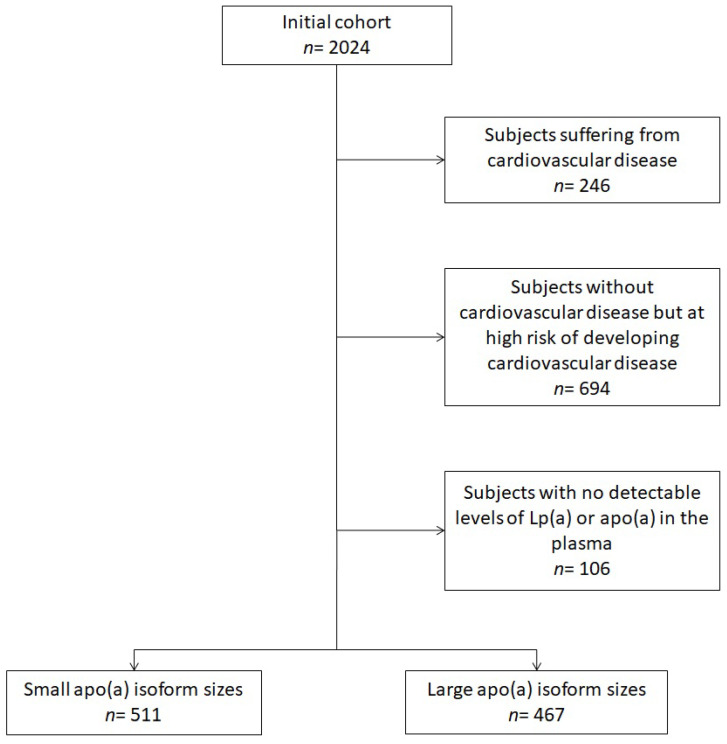
Flow-chart resuming the selection criteria applied to the full BHS cohort to select the sub-cohort.

For the purpose of the current analysis, from the entire 2012 BHS sample dataset, we excluded subjects suffering from CVD (i.e., subjects who had previously experienced CHD, minor or major stroke, peripheral obstructive artery disease), subjects at high risk for CVD (i.e., patients affected by heterozygous familial hypercholesterolemia (HeFH), patients with uncontrolled severe hypertension, pharmacologically treated type 2 diabetes mellitus (T2DM), severe chronic kidney disease, severe obesity) and subjects with no detectable levels of Lp(a) or apo(a) in the plasma. The remaining participants (462 men and 516 women) were divided into two groups depending on whether they had either small apo(a) isoform sizes or large apo(a) isoform sizes (Figure 1).

In accordance with the BHS protocol, each individual was evaluated with a thorough assessment of personal and family history (paying particular attention to lifestyle, smoking status, dietary habits and pharmacological treatments), a physical examination (including anthropometric data), resting blood pressure (BP) and heart rate. Fasting blood samples and 12-lead electrocardiograms (Minnesota-coded) were also collected [10]. Waist circumference (WC) was measured as the narrowest body diameter between the arcus costarum and the crista iliaca. Systolic (SBP) and diastolic (DBP) BP were measured three times at 1-min intervals with a standard sphygmomanometer (Omron M3 Intellisence), while the patient was seated and after a 5-min quiet rest. Three BP measurements were averaged and used as study variables [11].

### 2.2. Laboratory Analyses

Laboratory analyses were performed on venous blood sampled from the basilic vein after a 12-h overnight fasting. The following laboratory parameters were determined by trained personnel with standardized methods [12]: total cholesterol (TC), high-density lipoprotein cholesterol (HDL-C), triglycerides (TG), LDL-C, fasting plasma glucose (FPG), gamma-glutamyl-transferase (gGT), alanine aminotransferase (AST), aspartate aminotransferase (ALT), and creatinine and serum uric acid (SUA). Glomerular filtration rate (eGFR) was estimated by the Chronic Kidney Disease Epidemiology Collaboration (CKD-EPI) formula [13]. Serum levels of Lp(a) were determined by a sandwich enzyme-linked immunosorbent assay method (Macra-Lp(a); SDI, Newark, DE), that is an isoform-independent immunological assay considered the gold standard by the International Federation of Clinical Chemistry and Laboratory Medicine (IFCC) and approved by the World Health Organization (WHO) to measure Lp(a) [14]. Apolipoprotein isoform analyses were performed by electrophoresis in 1.5% SDS-agarose gels followed by immunoblotting and revealed by chemiluminescence according to the method of Langer et al. [15], being a reproducible analytical method that allows determining apo(a) phenotype with good specificity and sensibility. Because of the high degree of apo(a) polymorphism, apo(a) isoforms were classified into two subgroups on the basis of a previously identified cut-off [16]. The low-MW group comprised all isoforms with 14–25 Kringle-IV repeats, whereas the high-MW group included isoforms with a number of Kringle-IV repeats ≥26. This cut-off seems to distinguish the apo(a) isoforms associated with a higher atherothrombotic predisposition [17].

### 2.3. Arterial Stiffness Evaluation

Arterial stiffness was noninvasively evaluated by the use of the Vicorder^®^ instrument (Skidmore Medical Ltd., Bristol, UK) that is a commercially available validated, operator-independent device, previously used in other epidemiological studies [18,19].

CfPWV was measured by placing a neck cuff over the right carotid artery and a second cuff around the right thigh. As per the manufacturer’s recommendations, path length was obtained by recording the distance between the cuffs with the use of a tape measure. The cuffs were simultaneously inflated to 65 mmHg and the transit time (TT) was derived from the simultaneous acquisition of the carotid and femoral waveforms. Waveforms were visually inspected and a minimum of three good quality waveforms were captured. Two recordings with a difference in cfPWV of ≤0.5 m/s were accepted and averaged [20].

Pulse Wave Analysis (PWA) was measured by Vicorder^®^ with a specific algorithm deriving the central blood pressure curve from the radial pressure waveform [21].

### 2.4. Ankle Brachial Index

The measurements protocol and ankle-brachial index (ABI) calculation followed the American Heart Association (AHA) guidelines and the guidelines of the European Society of Cardiology (ESC) and the European Society of Hypertension (ESH) [22,23]. ABI was assessed by the use of Vicorder^®^ on the right and left sides, by placing pneumatic cuffs on the upper arms and lower legs (above the ankles). The cuffs were inflated to 200 mmHg simultaneously to occlude the brachial and tibial arteries. BP were taken at the point of the pulse returning at both sites as the cuffs slowly deflated and two measurements were recorded and averaged for each side. Three measures were taken for each side and a mean was taken only if the difference in sequential brachial and ankle recordings was >5 mmHg. All measures were made by two vascular technicians, with an intra-class correlation of 0.65 [24].

In agreement with the international recommendations, the ABI in each leg was calculated by dividing the highest pressure between the posterior tibial and dorsalis pedis arteries by the highest arm pressure. The lowest ABI amongst the two legs was considered as a study variable [25].

### 2.5. Statistical Analysis

All data were collected in a specific database. Categorical data were expressed as absolute number and percentage. A Kolmogorov-Smirnov normality test was performed to determine whether continuous data were normally distributed or not. A Levene test was performed to check the homogeneity of variances between the pre-specified groups. Normally distributed variables were compared by the Student’s *t*-test, while the non-normally distributed variables were compared by the Mann-Whitney-U test. Categorical variables were compared using the chi-square test. A multiple linear stepwise regression was carried out in order to detect the significant predictor of cfPWV in the entire population cohort and in the pre-specified subgroups. All statistical tests were two-sided and done at <0.05 significance level. Statistical analyses were done using the Statistical Package for Social Sciences (SPSS) version 25.0 for Windows (IBM Inc., Chicago, IL, USA).

The current analysis complied with the Strengthening the Reporting of Observational Studies in Epidemiology (STROBE) guidelines, and with the broader Enhancing the QUAlity and Transparency Of health Research (EQUATOR) guidelines [26].

## 3. Results

The main characteristics of the subjects included in the analysis were resumed in Table 1.

The pre-specified groups were balanced for sex, smoking habit and family history of early CVD (*p* > 0.05).

The main clinical and haemodynamic characteristics of the population sample were reported by apo(a) isoform size in Table 2.

The pre-defined groups were overall well balanced with respect to most of the clinically relevant characteristics. However, subjects with larger apo(a) isoform sizes had significantly lower cfPWV and higher values of SBP and aortic BP compared to the other group (*p* < 0.05). The most relevant laboratory characteristics of the population sample were reported by apo(a) isoform size in Table 3. The pre-defined groups were overall well balanced with respect to most of the clinically relevant characteristics. However, subjects with larger apo(a) isoform sizes had significantly higher levels of Lp(a) compared to the other group (*p* < 0.05).

In the entire population cohort, cfPWV was predicted by systolic blood pressure (SBP), serum levels of HDL-C, TG and sex, higher HDL-C serum levels and female sex being associated to lower values of cfPWV (Table 4).

In subjects with smaller apo(a) isoform sizes, predictors of cfPWV were age, SBP, sex and serum levels of HDL-C, higher HDL-C serum levels and female sex being associated with lower values of cfPWV (Table 4). In subjects with larger apo(a) isoform sizes, cfPWV was predicted by age, SBP, serum levels of Lp(a) and sex, female sex being associated to lower values of cfPWV (Table 4).

## 4. Discussion

In past decades, high serum levels of Lp(a) were considered a mainly genetically determined risk factor for the development of CVD, being only marginally influenced by some poorly tolerated lipid-lowering drugs [27,28]. For this reason, little attention was p aid to this lipid fraction for a long time, until the development of innovative drugs able to dramatically reduce the synthesis of Lp(a) in humans has recently drawn attention again [29,30].

Serum levels of Lp(a) are linearly associated to the risk of developing CVD, as has been recently shown by an individual-patient data meta-analysis including 29.069 patients [31]. At the same time, a large meta-analysis of 40 studies involving 11,396 patients and 46,938 controls concluded that individuals with smaller apo(a) isoforms have a higher relative risk (RR) of developing coronary heart disease (CHD) (RR = 2.08, 95% CI: 1.67 to 2.58) compared to individuals with larger apo(a) isoforms (intending 22 or fewer KIV type 2 repeats versus >22 repeats, or analogously an apo(a) molecular weight of <640 kDa versus ≥640 kDa) [32]. In effect, according to our observations, the inverse relationship between apo(a) size and serum concentrations of Lp(a) (i.e., low KIV copy number associates to high Lp(a) concentrations and vice versa) [33] was robust in the BHS, subjects with lower Lp(a) levels being more likely to have higher apo(a) sizes.

Even though Lp(a) levels have already been demonstrated to be independent predictors of CV mortality in the long term in the BHS population cohort [34], the aim of the present analysis was to investigate whether Lp(a) serum concentrations could also predict early vascular aging as measured by cfPWV, which is a non-expensive, non-invasive and reliable predictor of CV events. It should be acknowledged that different predictors for arterial stiffness were found in the historical cohort of the BHS by considering inclusion and exclusion criteria other than those used for the present analysis [35,36]. However, Lp(a) has never been included in those predictive models, so it remains uncertain whether or not it would have affected the results.

According to our findings, the serum concentration of Lp(a) significantly predicts vascular aging only in individuals with large apo(a) isoform sizes, while its predictive value is lost in individuals with smaller apo(a) isoform sizes and in general population. The clinical relevance of these observations is critical and has to be interpreted in light of the results from an earlier updated meta-analysis of 19 studies, concluding that participants with high cfPWV by 1 standard deviation (SD), 1 m/s, and cutoff points have a high pooled relative risk for CVD events (1 m/s: 1.12, 95% CI 1.07–1.18) and CVD mortality (1 m/s: 1.09, 95% CI 1.04–1.14) [37].

Clearly, the present analysis has several strengths, which provide adequate external validity for the obtained results, and also some limitations that should be acknowledged while suggesting further research directions. First, the BHS population is ethnically homogenous, which essentially rules out population stratification bias, even though it implies that the results may not be necessarily generalized to individuals of any ancestry other than European. The application of strict inclusion and exclusion criteria on the one hand minimized the risk of potential biases, but on the other leaded to relatively small subgroups (even though they were still representative of the whole population of Brisighella). It must also be emphasized that our results refer to a well-characterized cohort of individuals free from CVD and that, as per the study’s design, individuals with mild-to-moderate estimated CV risk and in pharmacological treatment with drugs potentially affecting cfPWV (e.g., antihypertensive and/or lipid-lowering drugs) were not excluded. From a laboratory point of view, the use of electrophoresis followed by immunoblotting might not be the gold standard for apo(a) analysis. However, this method has also been used in the large landmark Heart Protection Study 2-Treatment of HDL to Reduce the Incidence of Vascular Events (HPS2-TRHIEVE) Trial [38], and our results are in agreement with previous literature associating smaller apo(a) isoforms with worse cardiovascular health [32,33].

We are confident that these results will be useful to plan further research investigating the effects of emerging drugs affecting Lp(a) serum levels in individuals with smaller or larger apo(a) isoform sizes. Of course, additional data from different and larger population cohorts are needed to confirm our preliminary observations.

## 5. Conclusions

In our subpopulation sample, Lp(a) did not predict cfPWV. However, in subjects with large apo(a) isoform sizes, Lp(a) was a significant predictor of arterial stiffness.

## Figures and Tables

**Table 1:** Main characteristics of the study population in the entire cohort and by apo(a) isoform sizes.

	Smaller Apo(a) Isoform Size (N. 511)			Larger Apo(a) Isoform Size (N. 467)		
Sex	**Men**	**Women**		**Men**	**Women**	
	247 (53.5%)	215 (46.5%)		264 (51.2%)	252 (48.8%)	
Smoking habit	**No-Smokers**	**Current smokers**	**Ex-smokers**	**No-Smokers**	**Current smokers**	**Ex-smokers**
	278 (54.8%)	131 (25.8%)	98 (19.3%)	269 (57.8%)	119 (25.6%)	77 (16.6%)
Family history of early CVD	**No**	**Yes**		**No**	**Yes**	
	480 (93.9%)	31 (6.1%)		432 (93.5%)	35 (7.5%)	

CVD = Cardiovascular disease; N = Number of subjects.

**Table 2:** Clinical and haemodynamic characteristics of the population sample, reported by apo(a) isoform size.

Parameters	Smaller Apo(a) Isoform Size (N. 511)		Larger Apo(a) Isoform Size (N. 467)	
	Mean	Standard Deviation	Mean	Standard Deviation
Age (years)	56.9	10.9	60.1	10.1
Waist circumference (cm)	91.8	14.0	92.8	12.9
Body mass index (Kg/m^2^)	26.6	4.5	26.9	4.6
Systolic blood pressure (mmHg)	137.4	11.3	142.8 *	9.6
Diastolic blood pressure (mmHg)	73.1	5.2	73.8	4.8
Heart Rate (bpm)	63.3	12.1	63.7	11.0
Aortic blood pressure (mmHg)	134.4	10.6	139.8 *	9.7
Aortic pulse pressure (mmHg)	60.20	8.7	63.7	10.2
Augmentation Index	24.3	9.8	26.0	8.1
Cardiac output (L/min)	6.6	2.3	7.2	2.3
Stroke volume (mL)	106.8	13.0	110.8	18.1
Anke brachial index	1.13	0.16	1.13	0.15
Carotid femoral pulse wave velocity (m/s)	9.39	1.68	8.49 *	2.41

* *p* < 0.05 versus smaller apo(a) isoform sizes. N = Number of subjects.

**Table 3:** Most clinically relevant laboratory characteristics of the population sample, reported by apo(a) isoform size.

Parameters	Smaller Apo(a) Isoform Size (N. 511)		Larger Apo(a) Isoform Size (N. 467)	
	Mean	Standard Deviation	Mean	Standard Deviation
Fasting plasma glucose (mg/dL)	94.6	15.0	96.6	15.1
Total cholesterol (mg/dL)	213.5	21.8	221.1	17.4
Triglycerides (mg/dL)	118.3	70.8	122.5	77.2
HDL-cholesterol (mg/dL)	52.9	5.3	52.2	5.6
LDL-cholesterol (mg/dL)	137.5	17.1	145.1	15.6
Apolipoprotein B (mg/dL)	89.6	20.6	92.7	19.5
Apolipoprotein AI (mg/dL)	155.5	28.4	155.7	26.9
Lipoprotein (a) (mg/dL)	34.4	6.4	29.5 *	6.5
Serum uric acid (mg/dL)	5.2	1.3	5.2	1.2
eGFR (mL/min)	84.2	5.2	81.9	4.9

eGFR = Estimated glomerular filtration rate; HDL = High-density lipoprotein; LDL = Low-density lipoprotein; N = Number of subjects. * *p* < 0.05 versus smaller apo(a) isoform sizes.

**Table 4:** Multiple linear regression models predicting cfPWV in the entire population cohort and in the pre-defined subpopulation groups.

	Variables	Beta	RR	95% Confidence Intervals		*p*
				Lower Limit	Upper Limit	
**Entire cohort (N. 978)**						
Model 1 (R = 0.397)	Age	0.397	0.254	0.146	0.362	<0.001
Model 2 (R = 0.423)	Age SBP	0.312 0.170	0.242 0.118	0.133 0.111	0.352 0.125	<0.001 <0.001
Model 3 (R = 0.432)	Age SBP HDL-C	0.338 0.182 −0.92	0.246 0.119 −0.114	0.137 0.112 −0.205	0.355 0.126 −0.024	<0.001 <0.001 0.004
Model 4 (R = 0.437)	Age SBP HDL-C TG	0.337 0.178 −0.116 0.070	0.246 0.119 −0.118 0.103	0.136 0.112 −0.108 0.001	0.355 0.126 −0.029 0.005	<0.001 <0.001 <0.001 0.027
Model 5 (R = 441)	Age SBP HDL-C TG Sex (W versus M)	0.347 0.180 −0.143 0.073 −0.64	0.247 0.119 −0.123 0.103 −0.027	0.138 0.112 −0.034 0.001 −0.054	0.057 0.026 −0.012 0.005 −0.010	<0.001 <0.001 <0.001 0.022 0.042
**Smaller apo(a) isoform size (N. 511)**						
Model 1 (R = 0.404)	Age	0.404	0.243	0.134	0.351	<0.001
Model 2 (R = 0.423)	Age SBP	0.336 0.144	0.235 0.112	0.126 0.014	0.345 0.169	0.005 0.013
Model 3 (R = 0.434)	Age SBP Sex (W versus M)	0.342 0.135 −0.098	0.236 0.111 −0.331	0.127 0.004 −0.602	0.346 0.159 −0.060	0.004 0.017 <0.001
Model 4 (R = 0.444)	Age SBP Sex (W versus M) HDL-C	0.377 0.142 −0.135 −0.107	0.240 0.112 −0.457 −0.113	0.130 0.004 −0.748 −0.202	0.350 0.119 −0.167 −0.024	<0.001 0.011 <0.001 0.002
**Larger apo(a) isoform size (N. 467)**						
Model 1 (R = 0.363)	Age	0.363	0.207	0.144	0.372	<0.001
Model 2 (R = 0.397)	Age SBP	0.271 0.185	0.208 0.106	0.128 0.011	0.359 0.235	<0.001 <0.001
Model 3 (R = 0.414)	Age SBP Sex (W versus M)	0.302 0.205 −0.126	0.208 0.106 0.109	0.132 0.013 −0.241	0.364 0.168 −0.017	<0.001 <0.001 0.004
Model 4 (R = 0.423)	Age SBP Lp(a) Sex (W versus M)	0.305 0.216 0.189 −0.134	0.208 0.106 0.103 0.109	0.133 0.015 0.012 −0.242	0.365 0.139 0.218 −0.011	<0.001 <0.001 0.004 0.009

HDL-C = High-density lipoprotein cholesterol; Lp(a) = Lipoprotein(a); M = Men; N = Number of subjects; SBP = Systolic blood pressure; W = Women.

## Data Availability

Data supporting findings of this analysis are available from the University of Bologna. Data are available from the authors with the permission of the University of Bologna.

## Abbreviations

ABI = Ankle-brachial index; AHA = American Heart Association; apo(a) = Apolipoprotein(a); ALT = Aspartate aminotransferase; AST = Alanine aminotransferase; BHS = Brisighella Heart Study; BP = Blood pressure; cfPWV = carotid-femoral pulse wave velocity; CHD = Coronary heart disease; CI = Confidence interval; CKD-EPI = Chronic Kidney Disease Epidemiology Collaboration; CV = Cardiovascular; CVD = Cardiovascular disease; Cys = Cysteine; DBP = Diastolic blood pressure; eGFR = Estimated glomerular filtration rate; EQUATOR = Enhancing the QUAlity and Transparency Of health Research; ESC = European Society of Cardiology; ESH = European Society of Hypertension; FPG = Fasting plasma glucose; GFR = Glomerular filtration rate; gGT = Gamma-glutamyl-transferase; HDL-C = High-density lipoprotein cholesterol; HeFH = Heterozygous familial hypercholesterolemia; KIV = Kringle IV; KIV1 = Kringle IV type 1; KIV2 = Kringle IV type 2; KIV3–10 = Kringles IV types 3–10; LDL-C = Low-density lipoprotein cholesterol; Lp(a) = Lipoprotein(a); M = Men; N = Number of subjects; PWA = Pulse wave analysis; RR = Relative risk; SBP = Systolic blood pressure; STROBE = Strengthening the Reporting of Observational Studies in Epidemiology; SUA = Serum uric acid; T2DM = Type 2 diabetes mellitus; TC = Total cholesterol; TG = Triglycerides; TT = Transit time; W = Women; WC = Waist circumference.

## References

[1] Peng J., Liu M.M., Liu H.H., Xu R.X., Zhu C.G., Guo Y.L., Wu N.Q., Dong Q., Cui C.J., Li J.J. (2021). Lipoprotein (a)-mediated vascular calcification: Population-based and in vitro studies. Metabolism.

[2] Ruscica M., Sirtori C.R., Corsini A., Watts G.F., Sahebkar A. (2021). Lipoprotein(a): Knowns, unknowns and uncertainties. Pharmacol. Res..

[3] van der Hoek Y.Y., Wittekoek M.E., Beisiegel U., Kastelein J.J., Koschinsky M.L. (1993). The apolipoprotein(a) kringle IV repeats which differ from the major repeat kringle are present in variably-sized isoforms. Hum. Mol. Genet..

[4] Huang Z., Yang Y., Lu J., Liang J., He Y., Yu Y., Huang H., Li Q., Wang B., Li S. (2021). Association of Lipoprotein(a)-Associated Mortality and the Estimated Glomerular Filtration Rate Level in Patients Undergoing Coronary Angiography: A 51,500 Cohort Study. Front. Cardiovasc. Med..

[5] Liu J., Liu L., Wang B., Chen S., Liu B., Liang J., Huang H., Li Q., Lun Z., Ying M. (2021). Coronary Artery Disease: Optimal Lipoprotein(a) for Survival-Lower Is Better? A Large Cohort with 43,647 Patients. Front. Cardiovasc. Med..

[6] Brosolo G., Da Porto A., Bulfone L., Vacca A., Bertin N., Colussi G., Cavarape A., Sechi L.A., Catena C. (2021). Plasma Lipoprotein(a) Levels as Determinants of Arterial Stiffening in Hypertension. Biomedicines.

[7] Sorokin A., Kotani K. (2015). Lipoprotein(a) and Arterial Stiffness Parameters. Pulse.

[8] Cicero A.F.G., Gitto S., Fogacci F., Rosticci M., Giovannini M., D’Addato S., Andreone P., Borghi C., Brisighella Heart Study Group Medical and Surgical Sciences Dept., University of Bologna (2018). Fatty liver index is associated to pulse wave velocity in healthy subjects: Data from the Brisighella Heart Study. Eur. J. Intern. Med..

[9] Cicero A.F.G., Fogacci F., Rizzoli E., D’Addato S., Borghi C. (2021). Long-Term Impact of Different Triple Combination Antihypertensive Medications on Blood Pressure Control, Metabolic Pattern and Incident Events: Data from the Brisighella Heart Study. J. Clin. Med..

[10] Coppola P., Cicero A.F.G., Fogacci F., D’Addato S., Bacchelli S., Borghi C., On Behalf of the Brisighella Heart Study Group (2021). Laboratory and Instrumental Risk Factors Associated with a Sudden Cardiac Death Prone ECG Pattern in the General Population: Data from the Brisighella Heart Study. J. Clin. Med..

[11] Cicero A.F.G., Fogacci F., Giovannini M., Grandi E., D’Addato S., Borghi C., Brisighella Heart Study Group (2019). Interaction between low-density lipoprotein-cholesterolaemia, serum uric level and incident hypertension: Data from the Brisighella Heart Study. J. Hypertens..

[12] Cicero A.F., Rosticci M., Bove M., Fogacci F., Giovannini M., Urso R., D’Addato S., Borghi C., Brisighella Heart Study Group (2017). Serum uric acid change and modification of blood pressure and fasting plasma glucose in an overall healthy population sample: Data from the Brisighella heart study. Ann. Med..

[13] Levey A.S., Stevens L.A., Schmid C.H., Zhang Y.L., Castro A.F., Feldman H.I., Kusek J.W., Eggers P., Van Lente F., Greene T. (2009). A new equation to estimate glomerular filtration rate. Ann. Intern. Med..

[14] Dati F., Tate J.R., Marcovina S.M., Steinmetz A. (2004). International Federation of Clinical Chemistry and Laboratory Medicine, & IFCC Working Group for Lipoprotein(a) Assay Standardization. First WHO/IFCC International Reference Reagent for Lipoprotein(a) for Immunoassay—Lp(a) SRM 2B. Clin. Chem. Lab. Med..

[15] Langer C., Tambyrayah B., Nowak-Göttl U. (2013). Testing for apolipoprotein(a) phenotype using isoelectric focusing and immunoblotting technique. Methods Mol. Biol..

[16] Gazzaruso C., Garzaniti A., Buscaglia P., Bonetti G., Falcone C., Fratino P., Finardi G., Geroldi D. (1998). Apolipoprotein(a) phenotypes and their predictive value for coronary heart disease: Identification of an operative cut-off of apolipoprotein(a) polymorphism. J. Cardiovasc. Risk.

[17] Emanuele E., Lusignani L.S., Peros E., Montagna G., D’Angelo A., Montagna L., Geroldi D. (2004). Lipoprotein(a)-associated atherothrombotic risk in hemodialysis patients. Am. J. Nephrol..

[18] Parsons T.J., Sartini C., Ellins E.A., Halcox J.P.J., Smith K.E., Ash S., Lennon L.T., Wannamethee S.G., Lee I.M., Whincup P.H. (2016). Objectively measured physical activity, sedentary time and subclinical vascular disease: Cross-sectional study in older British men. Prev. Med..

[19] Müller J., Ewert P., Hager A. (2015). Increased aortic blood pressure augmentation in patients with congenital heart defects e a cross sectional study in 1125 patients and 322 controls. Int. J. Cardiol..

[20] Hickson S., Butlin M., Broad J., Avolio A.P., Wilkinson I.B., McEniery C.M. (2009). Validity and repeatability of the Vicorder apparatus: A comparison with the SphygmoCor device. Hypertens. Res..

[21] McGreevy C., Barry M., Bennett K., Williams D. (2013). Repeatability of the measurement of aortic pulse wave velocity (aPWV) in the clinical assessment of arterial stiffness in community welling older patients using the Vicorder device. Scand. J. Clin. Lab. Investig..

[22] Aboyans V., Criqui M.H., Abraham P., Allison M.A., Creager M.A., Diehm C., Fowkes F.G., Hiatt W.R., Jönsson B., Lacroix P. (2012). Measurement and interpretation of the ankle-brachial index: A scientific statement from the American Heart Association. Circulation.

[23] Williams B., Mancia G., Spiering W., Agabiti Rosei E., Azizi M., Burnier M., Clement D.L., Coca A., de Simone G., Dominiczak A. (2018). 2018 ESC/ESH Guidelines for the management of arterial hypertension: The Task Force for the management of arterial hypertension of the European Society of Cardiology and the European Society of Hypertension: The Task Force for the management of arterial hypertension of the European Society of Cardiology and the European Society of Hypertension. J. Hypertens..

[24] Ruscica M., Macchi C., Fogacci F., Ferri N., Grandi E., Rizzoli E., D’Addato S., Borghi C., Cicero A.F., Brisighella Heart Study Group (2020). Angiopoietin-like 3 and subclinical peripheral arterial disease: Evidence from the Brisighella Heart Study. Eur. J. Prev. Cardiol..

[25] Samba H., Guerchet M., Ndamba-Bandzouzi B., Kehoua G., Mbelesso P., Desormais I., Aboyans V., Preux P.M., Lacroix P. (2019). Ankle Brachial Index (ABI) predicts 2-year mortality risk among older adults in the Republic of Congo: The EPIDEMCA-FU study. Atherosclerosis.

[26] Simera I., Moher D., Hoey J., Schulz K.F., Altman D.G. (2010). A catalogue of reporting guidelines for health research. Eur. J. Clin. Investig..

[27] Sahebkar A., Reiner Ž., Simental-Mendía L.E., Ferretti G., Cicero A.F. (2016). Effect of extended-release niacin on plasma lipoprotein(a) levels: A systematic review and meta-analysis of randomized placebo-controlled trials. Metabolism.

[28] Fogacci F., Ferri N., Toth P.P., Ruscica M., Corsini A., Cicero A.F.G. (2019). Efficacy and Safety of Mipomersen: A Systematic Review and Meta-Analysis of Randomized Clinical Trials. Drugs.

[29] Cicero A.F.G., Bove M., Borghi C. (2018). Pharmacokinetics, pharmacodynamics and clinical efficacy of non-statin treatments for hypercholesterolemia. Expert Opin. Drug Metab. Toxicol..

[30] Strilchuk L., Fogacci F., Cicero A.F. (2019). Safety and tolerability of injectable lipid-lowering drugs: An update of clinical data. Expert Opin. Drug Saf..

[31] Willeit P., Ridker P.M., Nestel P.J., Simes J., Tonkin A.M., Pedersen T.R., Schwartz G.G., Olsson A.G., Colhoun H.M., Kronenberg F. (2018). Baseline and on-statin treatment lipoprotein(a) levels for prediction of cardiovascular events: Individual patient-data meta-analysis of statin outcome trials. Lancet.

[32] Erqou S., Thompson A., Di Angelantonio E., Saleheen D., Kaptoge S., Marcovina S., Danesh J. (2010). Apolipoprotein(a) isoforms and the risk of vascular disease: Systematic review of 40 studies involving 58,000 participants. J. Am. Coll. Cardiol..

[33] Kostner K.M., Kostner G.M. (2017). Lipoprotein (a): A historical appraisal. J. Lipid Res..

[34] Fogacci F., Cicero A.F., D’Addato S., D’Agostini L., Rosticci M., Giovannini M., Bertagnin E., Borghi C., Brisighella Heart Study Group (2017). Serum lipoprotein(a) level as long-term predictor of cardiovascular mortality in a large sample of subjects in primary cardiovascular prevention: Data from the Brisighella Heart Study. Eur. J. Intern. Med..

[35] Cicero A.F.G., Kuwabara M., Johnson R., Bove M., Fogacci F., Rosticci M., Giovannini M., D’Addato S., Borghi C., Brisighella Heart Study Group (2018). LDL-oxidation, serum uric acid, kidney function and pulse-wave velocity: Data from the Brisighella Heart Study cohort. Int. J. Cardiol..

[36] Ruscica M., Ferri N., Fogacci F., Rosticci M., Botta M., Marchiano S., Magni P., D’Addato S., Giovannini M., Borghi C. (2017). Circulating Levels of Proprotein Convertase Subtilisin/Kexin Type 9 and Arterial Stiffness in a Large Population Sample: Data from the Brisighella Heart Study. J. Am. Heart Assoc..

[37] Zhong Q., Hu M.J., Cui Y.J., Liang L., Zhou M.M., Yang Y.W., Huang F. (2018). Carotid-Femoral Pulse Wave Velocity in the Prediction of Cardiovascular Events and Mortality: An Updated Systematic Review and Meta-Analysis. Angiology.

[38] Parish S., Hopewell J.C., Hill M.R., Marcovina S., Valdes-Marquez E., Haynes R., Offer A., Pedersen T.R., Baigent C., Collins R. (2018). Impact of Apolipoprotein(a) Isoform Size on Lipoprotein(a) Lowering in the HPS2-THRIVE Study. Circ. Genom. Precis. Med..

